# Implementing mind mapping in small-group learning to promote student engagement in the medical diagnostic curriculum: a pilot study

**DOI:** 10.1186/s12909-024-05318-0

**Published:** 2024-03-26

**Authors:** Jieyu He, Bei Wu, Haiying Zhong, Junkun Zhan, Lanyan Zhu, Jie Zhang, Yi Zeng, Zhihong Li

**Affiliations:** 1 Department of Geriatrics, The Second Xiangya Hospital, Central South University, Changsha, Hunan China; 2 Hospital Management Department, Medical Education Office, Central South University, Changsha, Hunan China; 3 Department of Hematology, The Second Xiangya Hospital, Central South University, Changsha, Hunan China; 4 Department of Internal Medicine, The Second Xiangya Hospital, Central South University, Changsha, Hunan China; 5 Department of Gastroenterology, The Second Xiangya Hospital, Central South University, Changsha, Hunan China; 6 Academic Department, The Second Xiangya Hospital, Central South University, Changsha, Hunan China; 7 The Second Xiangya Hospital, Central South University, Changsha, Hunan China

**Keywords:** Physical diagnostics, Medical education, Mind map, Student engagement

## Abstract

**Background:**

Medical diagnostics is a pivotal bridge curriculum that receives much less attention from undergraduates in non-clinical medicine health profession programs with less student engagement and poor performance. Mind mapping is an active learning strategy for graphically presenting radiant thinking to culture clinical reasoning. The purpose of this study was to explore whether students’ comprehensive diagnostic skills are enhanced through increased student engagement by employing mind mapping.

**Methods:**

We implemented mind mapping in small-grouped workshops with 86 junior undergraduates from preventive medicine program, for physical diagnostic sessions including physical examination (PE) maneuver, electrocardiogram (ECG) interpretation and medical history collection. We also conducted assessments of the above skills, as well as online surveys regarding their expectation on this course, self-evaluation of mind mapping in teaching and the learning process of all the modules.

**Results:**

Group members employing mind mapping in all PE sessions obtained higher scores in the heart and lung systems during the PE maneuver exam. Similarly, groups that made more in-depth mind maps achieved higher scores on the ECG quiz. In addition, groups displaying mind maps for history taking from normal classes and reformed class exhibited greater completeness of medical history with both standardized patients and real patients, which was consistent with increased collection of accompanying symptoms. Mind mapping was valued by the majority of students for its benefits in terms of acquiring PE maneuver, theoretical knowledge, medical history collection and medical records writing, clinical reasoning, communication skills, sense of teamwork and cooperation, professionalism and humanistic literacy.

**Discussion:**

The visual feature of mind mapping evoked extensive behavioral engagement in all groups, as did cognitive and emotional engagement, as the majority of students expressed their willingness and affective reactions. In the short term, the positive feedbacks encourage growing engagement. The continuous benefits of mind mapping require long-term observation.

**Supplementary Information:**

The online version contains supplementary material available at 10.1186/s12909-024-05318-0.

## Introduction

Among bridge curricula in Chinese medical schools, the medical diagnostics curriculum is a pivotal module in transforming basic science and medical knowledge into critical thinking and diagnostic reasoning and comprises of physical and laboratory diagnostics [1, 2]. Its significance is usually more strongly perceived by medical undergraduates than by those from other health profession programs, as the latter of whom cannot be licenced for clinical practice. Moreover, observationally, far more students from non-medical programs are inclined to learn by rote, which could benefit the quick acquisition of theoretical knowledge, yet their performance of physical examination (PE) maneuver, auxiliary examination reading and medical history taking is commonly scored lower than that of medical undergraduates, consistent with active engagement. Moreover, the critical thinking from the former students developed in following clinical courses has been on fumes.

Based on the learning theory of constructivism, students’ active engagement is asserted to promote deep learning in both large classes and small-grouped settings [3, 4]. In medical education, teaching and learning approaches, such as problem-based learning, case-based learning, team-based learning and flipped classrooms, have been continuously reformed to support active learning, demonstrating advantages in the cultivation of clinical reasoning [5–9]. Moreover, strategies of deliberate practice involving more engagement in behavioral, cognitive and emotional aspects contribute to enhanced PE maneuver [10, 11]. In previous years, several of the abovementioned strategies were tested in diagnostic and clinical courses with undergraduates in preventive medicine program. Nevertheless, less engagement has been observed with harvest of relatively poor performance in assessments.

To overcome this challenge and to facilitate the acquisition of diagnostic skills, mind mapping was used in our study. Mind mapping is a graphic representation of radiant thinking that is demonstrated by displaying key ideas in the center and extending main branches in bright colors. Mind maps could be created on paper or via a number of software. Mind mapping has been viewed as an alternative strategy for retaining formation and fostering critical thinking in medical education by encouraging students to integrate information between disciplines and recognize relationships between concepts [12–16]. Making mind maps has been perceived as a time-consuming task [14], which implies maker involvement. On the other hand, mind mapping has been approved by medical undergraduates in a number of studies, who considered it a worthed time investment [14, 17, 18]. However, whether the strategy of mind mapping contributes to enhanced student engagement is unknown. Moreover, the effect of mind mapping in medical diagnostic course has not been reported. However, whether this approach could promote multiple diagnostic skills, including PE maneuver and history taking skills, remains unclear.

In our study, mind mapping was applied as a learning tool in the physical diagnostics section as an adjunct to didactic lectures in forms of small-group workshops for undergraduates in preventive medicine program. This pilot study intended to investigate the effect of this learning strategy on aspects of the PE maneuver, electrocardiogram (ECG) reading and medical history taking by encouraging student engagement.

## Method

### Participants

The study was conducted in the Second Xiangya Hospital affiliated with Central South University, China, from April 2021 to January 2022. The target population comprised 56 undergraduates in three normal classes and 30 in one reformed class both from third year of the Bachelor of preventive medicine program. All 86 undergraduates were placed in normal classes or reformed class based on their comprehensive scores (including grade average points) from the first year. Students who ranked among the highest in other programs with willingness to transfer major to this program and who ranked top in this program were grouped into reformed class, while other students in this program were divided in normal classes. During the sophomore year, all students were instructed with the same courses together in the same lecture halls. During the junior period, they were randomly classified into three groups (group 1, 2 and 3 from normal classes) and two groups (group 4 and 5 from reformed class) respectively. The compulsory courses they took were identical and included this medical diagnostic curriculum. All of them voluntarily participated in the research.

### Research design

The physical diagnostics section consisted of 10 sessions of 4-hour physical diagnostics covering the PE maneuver by system, auscultation on simulated models, ECG maneuver and reading, medical history collection and medical records writing, all of which were set as small-group workshops guided by the same instructor. 86 junior students in all groups were encouraged to create mind maps together by hand, or via software (e.g., XMind and PowerPoint) in the PE maneuver, ECG reading and medical history collection modules. Mind maps for each theme were required to be displayed in the forms of oral presentations or demonstrations in 30 minutes. Students preparing and presenting mind maps were rewarded for additional scores in general performance. Those groups that did not apply mind map were set as controls. Except for this strategy, all the teaching content and methods remained consistent in both mind-map and control groups.

### Assessment

The PE maneuver was evaluated once the course finished. ECG reading was tested at the end of the session, at which the mind map was presented and 2 weeks later, the same timepoint for the control groups. The completeness of medical history collection was assessed during practice with standardized patients (SPs) and real patients.

Online surveys were issued on SoJump (an online questionnaire platform) regarding expectation about this course and the benefits obtained throughout this course with the employment of mapping activities, as well as through evaluation of teaching and learning process on a Yes/No questions or 5-point Likert scale. They were collected separately before the semester, at the mid-exam and after the final-exam.

Mind maps were scored using the Mind Map Assessment Rubric (MMAR) as previously described [19]. The interrater reliability of the MMAR has been reported to be strong [19].

### Data analysis

The statistical analysis was conducted using IBM SPSS Statistics 25. The normality of the distribution of the test results was checked using the Shapiro Wilk test. A comparative study was performed using ANOVA or t-test for normal-distributed data. Otherwise, the Kruskal-Wallis test was applied. The results of the analysis were reported as medians/ interquartile ranges (for abnormal distribution). The level of significance was set at alpha = 0.05 for all the statistical tests.

## Results

### Consistent expectations between students and syllabus

Expectation on this curriculum was investigated ahead. From the most to the least, the acquisition of PE skills, theoretical knowledge, clinical reasoning, medical history collection, medical records writing and professionalism were mostly anticipated by almost two thirds of all the students (Table 1). It is indicated the students’ expectations were consistent with the course syllabus.

**Table 1:** Expectations on the diagnostics curriculum

Expectation	% (No. Votes)
PE maneuver	91.86% (79)
Theoretical knowledge	81.40% (70)
Clinical reasoning	80.23% (69)
Interview skills	69.77% (60)
Medical records writing	61.63% (53)
Professionalism and humanistic literacy	59.30% (41)
Doctor-patient Communication skills	52.33% (46)
Active learning	48.84% (42)
Teamwork & cooperation	46.51% (40)
Handling medical conflicts	40.7% (35)
Interest in other bridge and clinical courses	37.21% (32)

### Benefit of mind mapping in the PE maneuver

**Fig. 1:**
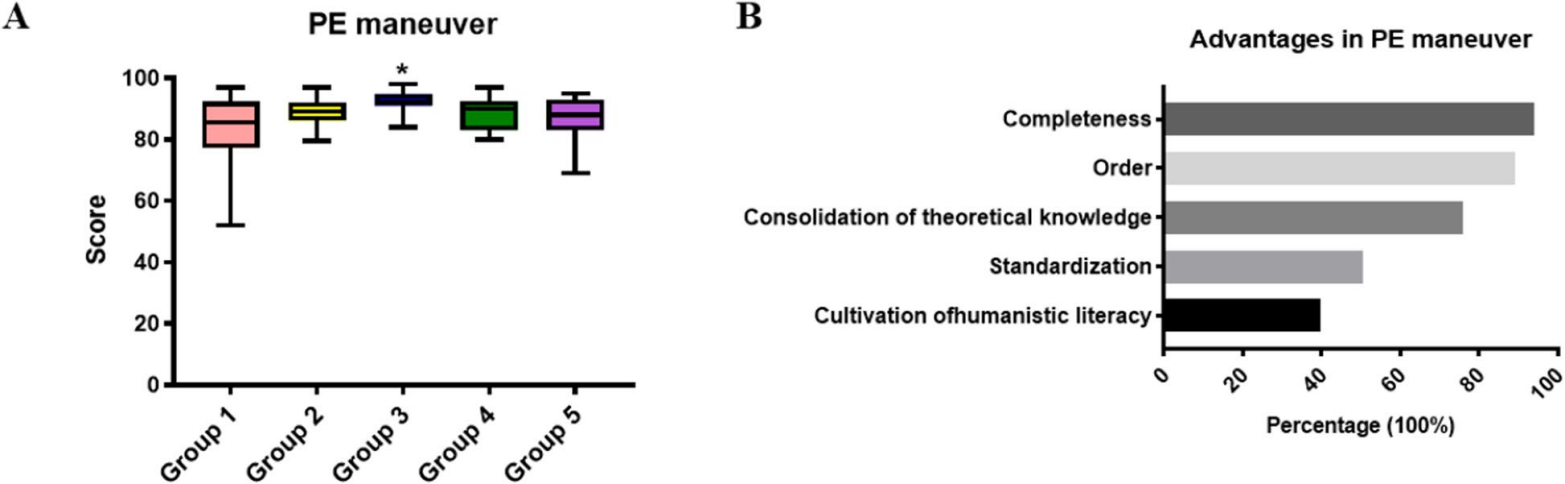
The benefit of mind mapping in the PE maneuver. Despite all groups displayed mind maps in sessions of separate PE maneuver, group 3 fully applied mind mapping in all sessions including general PE. Group 3 achieved the highest average score on the PE maneuver when compared with the other four groups (**A**). *, *p* < 0.05. **B** The advantages of mind mapping in PE maneuver are discussed and listed. PE: physical examination

First, mind mapping was applied in PE sessions. All groups displayed their mind maps on themes of head& neck, lung, heart, abdomen and nervous system, in the forms of oral presentations or combined with demonstrations at the start of the next session, except for group 3, whose members displayed mind map on general physical examination and demonstrated it in only 40 minutes fluently. Unsurprisingly, this group achieved significantly greater scores than did the other four groups on the evaluation of PE maneuver (*p* = 0.011), especially in parts of the lung (*p* = 0.011) and the heart (*p* = 0.011) (Fig. 1A, Table 2, Suppl Table 1). The advantages of mind mapping in PE maneuver were recognized as the acquisition of completeness and order of PE, the consolidation of theoretical knowledge and the standardization of PE as self-evaluated by more than 50% of all participants (Fig. 1B). The role of mind mapping in the cultivation of humanistic literacy was identified by nearly one third of participants (Fig. 1B).

**Table 2:** Comparison of the PE maneuver between the mind mapping group and control groups. The data are displayed as medians (P25, P75)

Score	MM group (Group 3)	Control (Group 1\2\4\5	*P* value
Head & Neck	90.5 (85.3, 92.8)	89.0 (84.6, 92.5)	0.862
Lung	94.0 (91.5, 96.5)	87.0 (81.8, 92.0)	0.012
Heart	93.3 (91.3, 95.3)	84.0 (81.8, 89.8)	0.012
Abdomen	92.8 (91.6, 96.9)	93.5 (89.3, 96.0)	0.192
Nervous system	91.5 (90.3, 95.8)	91.5 (88.5, 94.5)	0.862

### Benefit of mind mapping in ECG reading skill

**Fig. 2:**
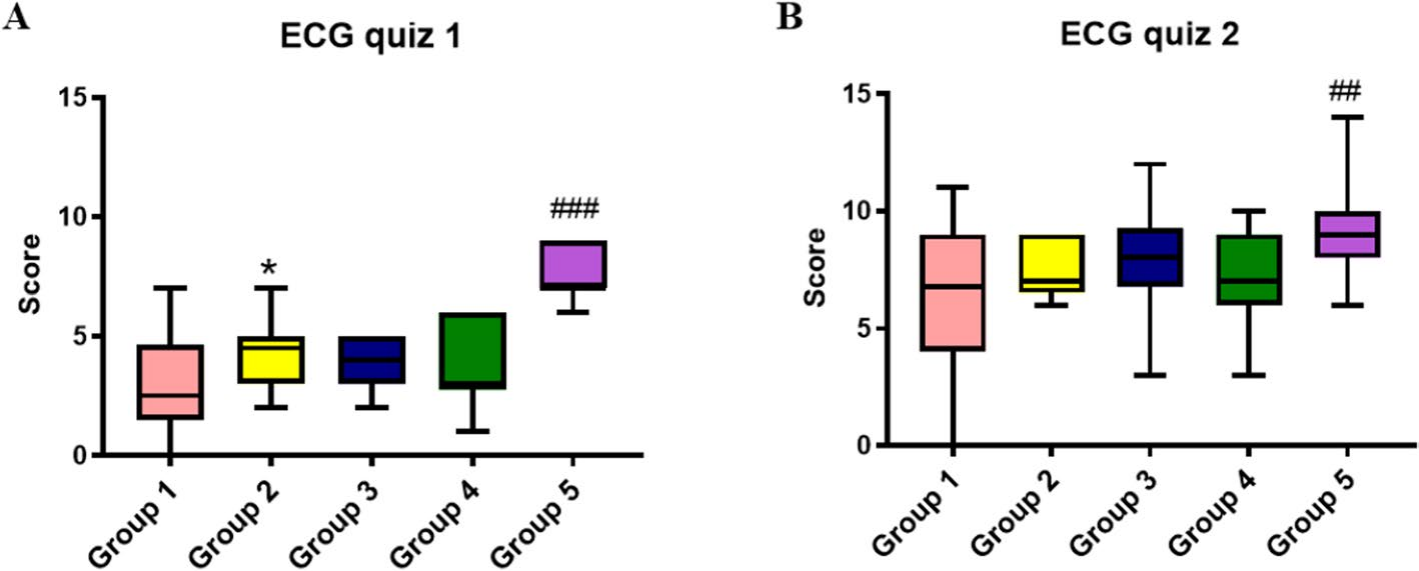
The benefit of mind mapping in ECG reading. Group 2 and Group 3 from the normal classes, and Group 5 from the reformed class employed mind mapping in the module for ECG reading. Group 1 from the normal classes and group 4 from the reformed class were included as controls. A quiz of 15 ECG images was given right at the end of that session (**A**) and 2 weeks later (**B**). * *p* < 0.05 compared with group 1; ## *p* < 0.01 compared with group 4; ### *p* < 0.005 compared with group 4

In the module of ECG reading, group 2 and group 3 in the normal classes and group 5 in the reformed class displayed their mind maps in the next session for the systemic structure of ECG interpretation. Then group 1 in the normal class and group 4 in the reformed class were set as the control groups in this module. The depth of mind maps by group 2 and group 5 was greater than that by group 3. A quiz of 15 ECG images was given right at the end of that session. Students in group 5 obtained significantly higher scores than did their peers from the reformed class. Similarly, scores in group 2 were significantly higher scores than those from the normal classes, while the average score in group 3 was comparatively greater than that in group 1 (Fig. 2A, Suppl Table 2). Additionally, these three groups were encouraged to polish their own mind maps collectively within 2 weeks. The same quiz was subsequently given to all groups again. On this occasion, group 5 still achieved higher score in reformed class, and even than all normal classes, while no significant difference was observed among the first three groups from the normal classes (Fig. 2B, Suppl Table 2).

### Benefit of mind mapping in medical history collection

**Fig. 3:**
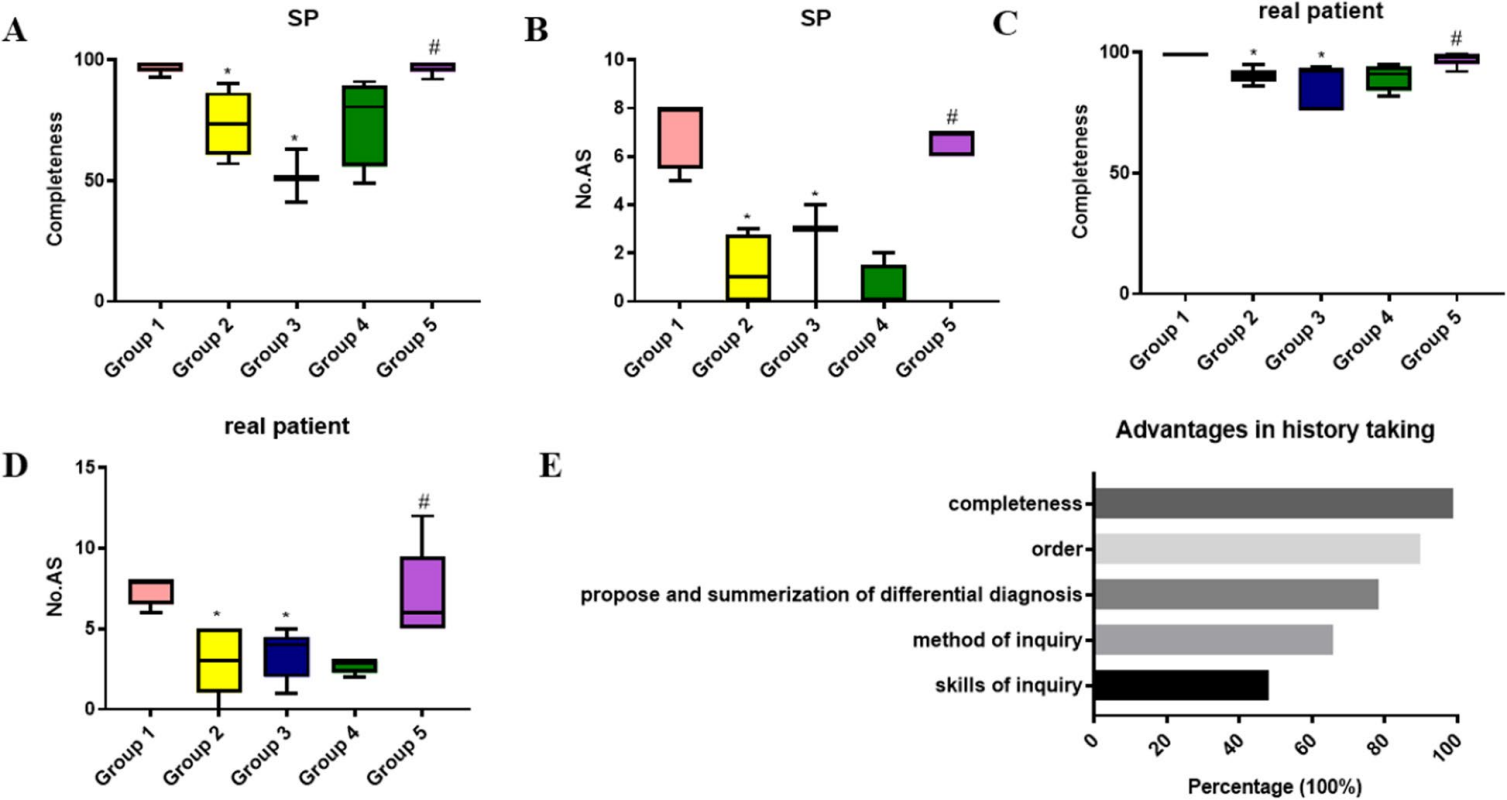
The benefit of mind mapping in history taking. At the start of this module, group 1 from the normal class, and group 5 from the reformed class employed mind mapping for five themes. Group 2, 3 from the normal classes and group 4 from the reformed class were controls. Then the practice of history taking was carried out with SPs and later real patients in groups of 3–5 students. The completeness of the history taking (**A**) and (**C**) and collected accompanying symptoms (**B**) and (**D**) were recorded during interview with SPs and real patients. **E** The advantages of mind mapping in history are discussed and listed. * *p* < 0.05 compared with group 1; # *p* < 0.05 compared with group 4. SP: standardized patient. No. AS: number of accompanying symptoms

In the module of medical history collection, all members from group 1 and group 5 selected five themes of common chief complaints (including chest pain, jaundice and dyspnea) for mind maps and presented them at the start of the same session as preview. Then group 2 and 3 in the normal classes and group 5 in the reformed class were set as controls. Practice with SPs and following real patients in the medical ward was carried out for all groups, all of which were observed and recorded by the instructor. The completeness of collected medical history and the number of accompanying symptoms were assessed. The two groups displayed mind maps for medical history collection spent less time completing medical history collection in almost all rounds of practice with SPs. Additionally, it was observed that these two groups accommodated themselves to practical scenes faster and with more confidence than did the control groups from corresponding class (es), as indicated with the greater completeness achieved and the greater number of accompanying symptoms proposed than did the corresponding control groups (Fig. 3A, B, Suppl Table 3). Similarly, the two mind-mapping groups obtained more complete medical history and raised more accompanying symptoms when communicating with real patients. Moreover, the three control groups gradually achieved greater completeness of medical history and an increased number of accompanying symptoms with real patients than that with SPs (Fig. 3C, D, Suppl Table 3). The advantages of mind mapping in medical history collection included the acquisition of completeness and order of proposal, the summarization of differential diagnosis, and the gain of method and skills of inquiry by more than 50% of all participants (Fig. 3E).

### Other benefits of mind mapping in this course

**Fig. 4:**
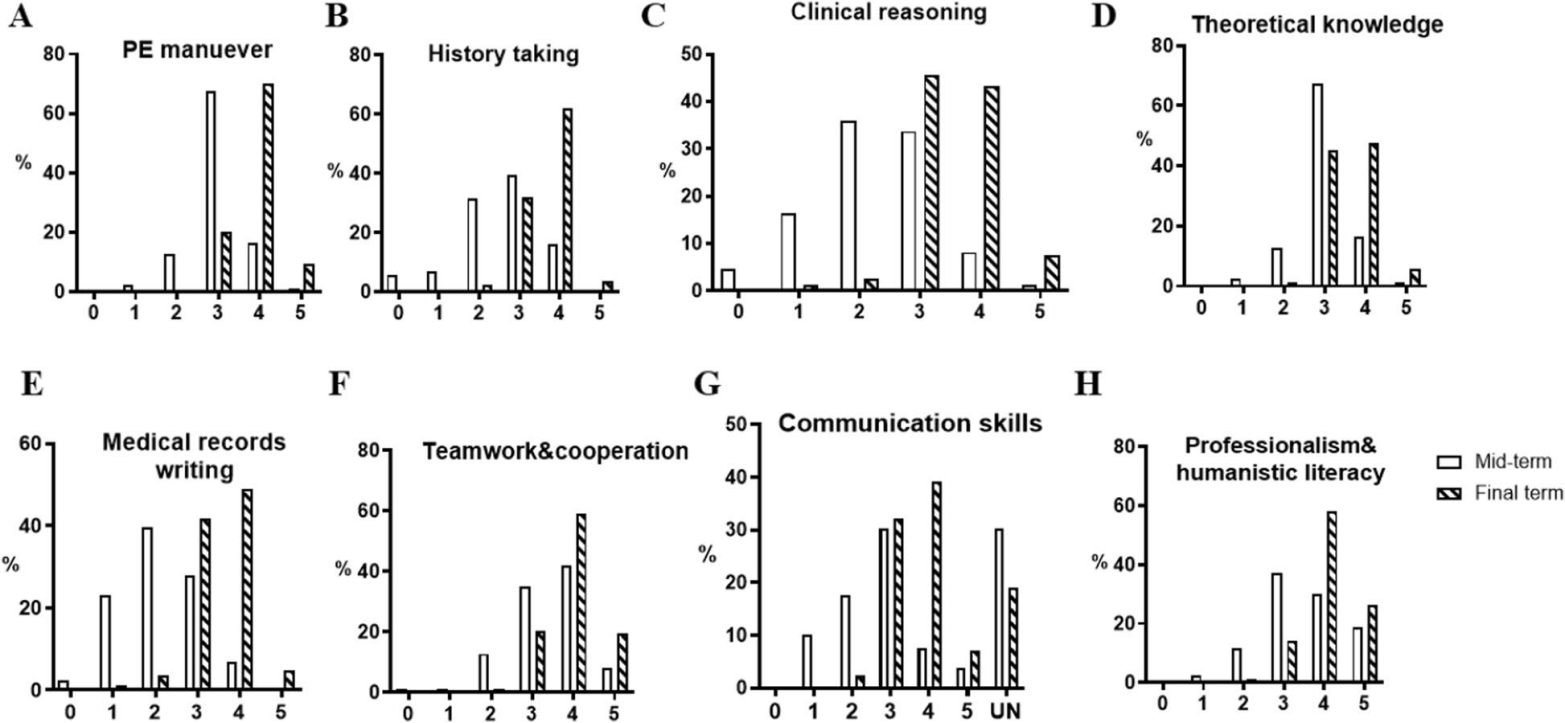
Additional benefits of mind mapping in physical diagnostics. The students self-assessed the benefits gained with mind mapping through this course at the mid-exam and final-exam timepoints. In addition to sustained enhancement in the acquisition of PE maneuver (**A**), history taking (**B**) and clinical reasoning (**C**), mind mapping contributed to enhanced theoretical knowledge (**D**), medical records writing (**E**), sense of teamwork and cooperation (**F**), communication skills (**G**), professionalism and humanistic literacy (**H**). PE: physical examination

In the modules of PE maneuver, every member in all groups positively asked for making mind maps and finally reached collaborative agreements on the sequence of this activity. In modules of ECG interpretation and medical history collection, the group members who made mind maps might discuss how to improve and to present their maps with the instructor. Furthermore, all groups provided science education on common chronic diseases (e.g. hypertension, diabetes, cirrhosis and Alzheimer’s disease) and even about the COVID-19 pandemic in forms of stageplay, live and animated videos in the extra-curriculum activity. Meanwhile, in our midterm and final-term survey, the response rate was over 97.6%. All the students in the five groups agreed that mind mapping was appropriate for the PE maneuver. The recognition of mind mapping in ECG readings was 100.0% in group 2 and group 3, and 93.3% in group 5 (Table 3). Acceptance in medical history collection was 100% in group 1 and group 5 (Table 3). As expected, mind mapping was recognized to consistently facilitate the acquisition of PE maneuver, theoretical knowledge, medical history collection and medical records writing, as well as improve clinical reasoning, communication skills, a sense of teamwork and cooperation, professionalism and humanistic literacy (Fig. 4A-H). As mind mapping has been employed in PE sessions since the beginning of the course, students rated general high on the acquisition of PE maneuver (Fig. 4A) and theoretical knowledge (Fig. 4D) at the midterm timepoint. They even rated sustained enhancement of the PE maneuver and theoretical knowledge after final-term. Meanwhile, since mind mapping was adopted in the medical history collection module after the midterm, self-evaluations of clinical reasoning, medical history collection, medical records writing and even communication skills were significantly greater at the the final-term than at the midterm (Fig. 4B, C, E-H). Additionally, when asking student what else has benefited, we received a number of responses, such as “I can get good grades in final exam”, “It makes case analysis easier”, “I can handle self-directed learning”, “I feel like falling in love with this course”, “I want to learn other medical courses”, “I am confident in future clinical rotation” and “My comprehensive abilities have increased”. Consistent self-evaluation has shown the omnifarious benefits of mind mapping in this course. Finally, most students in group 1 and group 4, and all members in other three groups favored continuing mind mapping in future courses (Table 3). Generally, mind mapping simultaneously evoked extensive behavioral engagement in all groups, as did cognitive and emotional engagement, as the majority expressed their willingness and affective reactions.

**Table 3:** Acceptance and favorability of mind mapping in different modules from five groups

Acceptance in Module	Group 1	Group 2	Group 3	Group 4	Group 5
PE maneuver	94.4%	100%	100%	100%	100%
ECG reading	/	100%	100%	/	93.3%
History taking	100%	/	/	/	100%
Favor	83.3%	100%	100%	93.3%	100%

## Discussion

This study investigated the implementation of mind mapping as a learning tool in medical diagnostic curriculum for preventive medicine undergraduates. Three surveys, one ahead of the curriculum and the other two at the midterm and final term, were distributed to students to explicate their expectations on this course, and to assess student satisfaction and the skills gained with mind mapping. The findings showed that mind mapping promoted the acquisition of comprehensive diagnostic skills, including PE maneuver, ECG reading and history taking skills, all of which were expected by students. Additionally, this study is the first to reported that mind mapping enhances student engagement, which directly contribute to improved performance in physical diagnostic education.

In the modules of PE maneuver, although all the groups displayed mind maps, only group 3 employed mind mapping in all the sessions, including general PE. The average scores achieved in students from group 3 were greater for the total PE maneuver, and especially in the lung and heart systems, than for the other four groups, despite potential variance in learning capacities between normal classes and reformed class. The students in the other four group, frequently made mistakes during percussion, palpation and auscultation. For example, some students might skip one or several auscultatory valve areas or become confused about the locations. In previous studies, medical undergraduates favored computer/internet-based [20–23], pocket card/checklist-assisted [24, 25], problem-based/hypothesis-driven learning [26, 27], simulation [28–30], peer-assisted learning [31, 32] and bedside teaching [33, 34] rather than traditional didactics with deliberate practice in the PE class, for reasons of time/space flexibility, explicit structure and memorableness, standardization of maneuvers, or real-world with human literacy emphasis, etc. The strategy of mind mapping in our study resembles peer-assisted preparation of shared pocket cards and checklists in the memorization of structural knowledge points. Therefore, the students’ comments concentrated on the advantages of a clear emphasis on theoretic knowledge, standardized structure and order of PE and organ-specific PE maneuver. The four groups that displayed mind maps on organ-specific PE maneuver they did not lose more in PE techniques, indicating that mind mapping did not support grasping precise techniques. It is implicated that displaying mind map on general PE could strengthen the memorization of all organ-specific PE maneuvers and their relationships, and could even cultivate human literacy to some extent, when students plan to avoid frequently having patients changing position during the whole PE. Additionally, we did not observe significant differences in the scores for various maneuvers between groups displaying mind maps in the forms of oral presentations and demonstrations. From another perspective, the extensive participation in making and displaying mind maps regarding PE maneuver supports student engagement. Firstly, the “reward” of additional scores in general performance might prompt general behavioral participation. Secondly, enhanced organ-specific PE maneuver might further prompt group 3 to challenge mind map of whole-body PE, whose process could be interpreted as cognitive engagement.

Unexpectedly, we have observed the benefit of mind mapping in ECG reading. ECG reading has been poorly performed at the undergraduate level [35–37]. Web-based deliberate practice and graphics-sequence memory methods provided by educators has proven effective in ECG interpretation [36, 37] Zeng et al provided students with a number of schematic diagrams of ECGs involving step-by-step analysis of the heart rate and rhythm, P wave, PR interval period, QRS wave, ST segment and T wave, as well as the QT interphase until U wave [37]. In this study, students from three groups drew their own reading schemes of both common and uncommon ECGs in one mind map while comprehending previous instruction and textbook knowledge. The mind maps of these groups varied in detail when comparing differences in ECG appearances, not only resembling the schematic diagrams by *Zeng* et al [37]. The depth of the mind maps was positively related to the ECG quiz scores among the mind-mapping groups. After displaying mind maps, students in group 5 made joint revisions to increase its depth (assessed by the MMAR [19]), indicating that they have progressed more quickly in ECG reading. Conversely, mind maps from another two groups were revised by individual students to limited depth, which could explain the variance in the second ECG quiz. The extent of ECG revision might also imply the variance in self-learning capability between normal classes and reformed class. Mind mapping facilitates a metacognitive process to integrate information by recognizing valid intra- and inter-relationships between concepts [38] of substantial ECG patterns. Wang S et al also reported that mind mapping made by students benefits divergent thinking as learners could logically correlate a variety of knowledge and overall depiction [39]. Basically, mind mapping benefits long-term memorization [15], while D‘Antonio et al and Wickramasinghe et al denied its benefit in short-term information retrieval [40, 41]. However, we demonstrated the effectiveness of mind mapping for the retention of short-term (3 hours) information. The studies by D‘Antonio et al and Wickramasinghe et al differ from this study in their design in that mind maps were taken as notes and were finished in class. Another study by *Bwaneh A* reported that teaching with mind mapping (displayed by instructors) increased immediate information retrieval but not long-term retention [42]. Although individuals in our study spent time out of class making mind maps, the maps benefited all the group members during the workshop. Furthermore, the unique property of mind maps in the added dimensions of pictures and colors appeals to diverse learning styles, such as linear or non-linear learners, visual learners, sequential learners and verbal learners [40, 43]. This finding could lead to the use of mind mapping as a novel learning tool for ECG instruction.

Medical history collection involves history taking skills and clinical reasoning. Clinical reasoning is the cognitive and metacognitive processes of analyzing knowledge related to a clinical situation or specific patients [44]. Critical thinking, a similar process in which knowledge is analyzed based on evidence and science, is a key skill integral for clinical reasoning. Among the evidence obtained from medical history during interviews with patients, accompanying symptoms, either negative or positive are essential for guiding differential diagnosis (critical thinking). A plethora of instructional and innovative approaches, including facilitation by scripts [45], role-play with SPs, virtual patients and real patients [46–49], and improvisational theater [50], all of which have benefited students in the early preclinical stage more from programs by helping them focus on interview skills but not being distracted by thinking about differential diagnoses [51]. As in our case, role-play is usually adopted in the module of medical history collection to practice interview skills, whilst the acquisition of critical thinking is more desirable through further clinical curricula and clerkship. The benefit of mind mapping in integrating critical thinking and problem solving skills, has been proven in resident training and clinical practice [52]. Similarly, mind mapping applied in another nursing program improved critical thinking and communication skills in undergraduates [53]. In our study, both self-evaluation questionnaires and assessment via a checklist during practice with SPs and real patients demonstrated consistently enhanced history taking skills. Surprisingly, an increased number of accompanying symptoms in the mind mapping groups during assessment indicated the emergence of critical thinking as supported by students’ feedback that mind mapping contributed to the propose and summarization of differential diagnosis. The additional benefit might be attributed to the feature of the mind map. Firs, the visual graphical feature of mind map confers the advantages of constructive learning, as the students explored to embed more items of accompanying symptoms in the mind maps as a representation of their critical thinking process [40]. Second, interpretation of the mind maps created by students reflect their gap between physicians in diagnostic thinking, which allows for the modification of teaching modalities to better suit the students [54].

Most importantly, the study strongly showed that student engagement in the domains of behavior, cognition and emotion, was enhanced by mind mapping. The effectiveness of mind mapping in retaining knowledge and critical thinking might be decided by the role of makers. As a teaching modality, mind maps displayed by instructors engage students better by liberating class time for discussion, but not improving long-term retention [42]. The mind maps drawn in class have been previously reported to distract students, while prepared mind maps projecting at the beginning or the end of the class facilitated establishment of the relationships between various topics and sub-topics [55]. As a learning modality, mind mapping engages students in class activities, which potentially demands more student involvement. When applied as note taking, it did not improve short-term retention [40, 41]. Unlike form of taking notes, in this study, mind maps were created out of class by students after reviewing knowledge points. However, this process is time-consuming even with the assistance of software. Although the time spent on making mind maps and rehearsing demonstrations were not assessed, they were recorded when the makers discussed them with the instructor or made joint revisions after class in all the modules. Additionally, the students presented omnifarious stage play and video shows in second-class activities. Active participation in the two dimensions well confirmed the domains of behavioral engagement [56]. In both the midterm and final-term surveys, the students approved of the benefits of mind mapping in three modules, and recognized unexpected benefits in terms of communication skills, a sense of teamwork and cooperation, professionalism and humanistic literacy, in addition to their interest in completing tasks involving mind maps during the course, all of which indicated cognitive engagement. The students’ affection for and commitment to employ mind mapping in other courses support emotional engagement. This finding is in line with a qualitative study focused on undergraduates’ perceptions of the application of mind mapping as a lifelong learning tool [57]. Evidently, this strategy demands and eventually evokes student engagement in all three dimensions, which can result in long-term benefits. Therefore, the comprehensive and continuous benefit of mind mapping in this curriculum must be evaluated.

### Limitations and strengths

This pilot study was conducted with undergraduates from non-clinical medicine health profession program. An advantage of this study is that we have obtained both objective assessment and subjective assessments of the usefulness and effectiveness of the presented strategy. A great boost in student engagement was observed during the course, but this was not obvious in the final scores on this curriculum and OSCE assessment 1 year later. An important limitation is the loss to follow-up observation in further clinical curricula and clerkship. It is likely that the students who are stuck to mind mapping will enjoy continuing benefits in clinical reasoning, interview skills, PE maneuver and even more abilities.

Future research should focus on several areas. First, how mind mapping impacts student engagement rather than motivation for material reward (additional scores) should be elaborated upon. Second, this strategy could be implemented in the same course but for medical undergraduates. It may be supposed that medical students would benefit more from mind mapping in their future resident training and clinical practice. In addition, the time span of implementation of mind mapping and observation is expected to increase with the involvement of more educators.

## Conclusion

Mind mapping was implemented in small-grouped workshops in the physical diagnostic curriculum. The results of the PE examination, ECG reading quiz and history taking assessment by checklist were used to demonstrate the effectiveness of the mind mapping strategy on improving undergraduates’ PE maneuver, ECG reading and history taking skills. Meanwhile, the students rated and discussed the usefulness of this strategy on each module. Surprisingly, we observed that mind mapping benefited students from developing clinical reasoning. All the benefits concluded by themselves reflected their recognition and favor of this learning strategy, which might partially explain their intensive engagement. Mind mapping ensures the involvement of students in active learning, as well as the transformation of instructors into more student-centered ones in their teaching. Future study programs are encouraged where the presented strategy can be applied.

### Supplementary Information

**Supplementary Material 1.**

## Data Availability

All the data generated or analyzed during this study are available upon the corresponding author on reasonable request. Informed consent was obtained from all participants.

## Abbreviations

PE: Physical examination
SP: Standardized patient
ECG: Electrocardiogram
OSCE: Objective structured clinical examination
AS: Accompanied symptoms
