# Supplementary material for: Implementing mind mapping in small-group learning to promote student engagement in the medical diagnostic curriculum: a pilot study
Source: BMC Med Educ. 2024 Mar 26;24:336. doi: 10.1186/s12909-024-05318-0 (PMC10967072; doi:10.1186/s12909-024-05318-0)
Supplement: Supplementary file 1 — Supplementary Material 1. [file 12909_2024_5318_MOESM1_ESM.pdf]

Supplementary Information (SI)

Implementing mind mapping in small-group learning to promote student engagement in the medical diagnostic curriculum: a pilot study

Jieyu He[1], Bei Wu[2], Haiying Zhong[3], Junkun Zhan[1], Lanyan Zhu[3], Jie Zhang[3*], Yi Zeng[1,4*], Zhihong Li[5]

1. Department of Geriatrics, the Second Xiangya Hospital, Central South University, Changsha, Hunan, China
2. Medical Education Office, Hospital Management Department, Central South University, Changsha, Hunan, China
3. Department of Internal Medicine, the Second Xiangya Hospital, Central South University, Changsha, Hunan, China
4. Academic Department, the Second Xiangya Hospital, Central South University, Changsha, Hunan, China
5. the Second Xiangya Hospital, Central South University, Changsha, Hunan, China

Correspondence should be addressed to:
Jie Zhang, Department of Internal Medicine, the Second Xiangya Hospital, Central South University, Changsha, Hunan, China, email: jiezhang@csu.edu.cn.
Yi Zeng, Academic Department, the Second Xiangya Hospital, Central South University, email: zengyi_xyneuro@csu.edu.cn.

Suppl Table 1 Comparison of the PE maneuver in the five groups. The data are displayed as medians (P25, P75).

Score	Group 3	Group 1	Group 2	Group 4	Group 5	*P* value
**Total**	92.5 (91.1, 94.9)	86.5 (82.8, 93.5)	89.0 (86.0, 92.0)	88.8 (82.3, 91.8)	86.3 (82.3, 89.0)	0.011
**Head&Neck**	90.5 (86.5, 92.5)	86.0 (82.5, 89.8)	88.0 (85.0, 90.0)	92.5 (91.3, 92.5)	85.0 (84.8, 89.8)	0.771
**Lung**	94.0 (92.0, 96.0)	88.0 (78.5, 93.0)	88.3 (83.5, 91.3)	91.0 (85.5, 91.5)	82.0 (82.0, 85.0)	0.771
**Heart**	93.3 (91.5, 95.3)	69.5 (53.0, 88.5)	88.8 (85.8, 90.5)	82.0 (81.5, 82.5)	89.0 (86.0, 91.0)	0.771
**Abdomen**	92.8 (91.8, 95.8)	93.5 (89.3, 96.0)	90.5 (83.8, 93.3)	88.5 (86.8, 88.8)	87.5 (78.3, 88.3)	0.771
**Nervous system**	90.5 (90.5, 94.5)	91.0 (89.5, 95.0)	90.0 (88.5, 93.5)	93.0 (92.5, 95.0)	93.0 (92.0, 94.0)	0.775

Suppl Table 2 Comparison of the ECG reading in the five groups. The data are displayed as medians (P25, P75).

Score	Group 1	Group 2	Group 3	*P* value	Group 4	Group 5	*P* value
**quiz 1**	2.5 (1.5, 4.6)	4.5 (3.0, 5.0)	4.0 (3.0, 5.0)	0.021	3.0 (2.9, 6.0)	7.0 (7.0, 9.0)	0.000
**quiz 2**	6.8 (4.0, 9.0)	7.0 (6.5, 9.0)	8.0 (6.8, 9.3)	0.179	7.0 (6.0, 9.0)	9.0 (8.0, 10.0)	0.009

Suppl Table 3 Comparison of the history taking in the five groups Scores of completeness and number of accompanying symptoms (AS) collected were recorded during history taking with standard patients (SPs) and real patients. The data are displayed as medians (P25, P75).

SP	Group 1	Group 2	Group 3	*P* value	Group 4	Group 5	*P* value
**Completeness score**	98.0 (97.0, 99.0)	73.5 (64.0, 83.0)	53.0 (46.0, 57.0)	0.010	80.5 (62.5, 88.0)	98.0 (98.0, 99.0)	0.016
**No. AS**	8.0 (6.0, 8.0)	1.0 (0, 2.5)	3.0 (1.5, 3.5)	0.013	0.0 (0, 1.0)	7.0 (6.0, 7.0)	0.016
**Real patient**							
**Completeness score**	99.0 (99.0, 99.5)	90.0 (90.0, 90.0)	92.0 (76.0, 93.0)	0.017	91.0(86.0, 93.5)	98.0 (98.0, 99.0)	0.032
**No. AS**	8.0 (7.0, 8.0)	3.0 (2.0, 5.0)	4.0 (3.0, 4.0)	0.017	3.0 (2.5, 3.0)	7.0 (5.0, 7.0)	0.016
